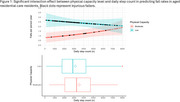# Rethinking the role of ambulatory activity in falls risk in aged residential care: influence of physical capacity and cognitive impairment

**DOI:** 10.1002/alz70858_100784

**Published:** 2025-12-25

**Authors:** Ríona Mc Ardle, Lynne Taylor, Silvia Del Din, Lynn Rochester, Ngaire Kerse, Jochen Klenk

**Affiliations:** ^1^ NIHR Newcastle Biomedical Research Centre, Newcastle University, Newcastle upon Tyne, Tyne and Wear, United Kingdom; ^2^ Translational and Clinical Research Institute, Newcastle University, Newcastle upon Tyne, Tyne and Wear, United Kingdom; ^3^ University of Auckland, Auckland, Auckland, New Zealand; ^4^ National Institute for Health and Care Research, Biomedical Research Centre, Newcastle upon Tyne, Tyne and Wear, United Kingdom; ^5^ Newcastle University, Translational And Clinical Research Institute, Newcastle upon Tyne, United Kingdom; ^6^ The University of Auckland, Auckland, New Zealand; ^7^ Ulm University, Ulm, Germany

## Abstract

**Background:**

Aged residential care (ARC) residents have 2‐4x greater fall risk than older community‐dwellers, leading to injuries, hospitalisation, and death. High prevalence of dementia and physical impairments exacerbate fall risk. Facilities often restrict residents’ ambulatory activity as a falls prevention measure but evidence linking ambulatory activity and falls rates in ARC is unclear. To address this risk‐reward conundrum presented by care staff, we aimed to explore the relationship between ambulatory activity and falls rate in ARC considering the influence of physical capacity and cognitive impairment.

**Method:**

Data from 281 ARC residents in New Zealand‐based Staying Upright randomised controlled trial. Participants demonstrated moderate cognitive impairment (Montreal Cognitive Assessment (mean±SD) 15±6). Step count was assessed via a lower‐back accelerometer for seven days at baseline. Falls were monitored for participants’ study duration (581±264 days). Physical capacity was classified as Moderate (*n* = 71) or Low (*n* = 205) using the Short Performance Physical Battery scores. Quasi‐Poisson generalised linear models assessed associations and interactions between steps, cognition, physical capacity, and falls rates. Relative risks of falls and fall‐related injuries were estimated for activity levels (2000, 4000, 6000 steps/day).

**Result:**

The Low physical capacity group had higher falls rates than Moderate (*p* = 0.001). Higher daily step count were linked with increased falls rate (*p* = 0.046), with a significant interaction (*p* = 0.036): the Moderate Group showed a positive association between steps and falls, while the Low group did not (Figure 1). Cognitive impairment was unrelated to steps or falls. Moderate‐capacity participants had a ∼23‐24% increased relative fall risk between activity levels, with only a ∼6% increase in injury risk. The Low‐capacity group had negligible changes (∼2.7‐3.9%) in relative risk across activity levels.

**Conclusion:**

Residents with higher physical capacity can take more falls‐free steps. While their falls risk increases slightly as activity levels increase, this is not at the expense of injuries. Low‐capacity residents face higher baseline fall risk and incidence of injurious falls but are less influenced by activity levels. Despite perceptions that cognitive impairment increases fall risk, no associations were found. These results challenge the practice of restricting ambulatory activity in aged care to prevent falls, highlighting the need for tailored interventions.